# Supplementary material for: Association between maternal vegetable intake during pregnancy and allergy in offspring: Japan Environment and Children’s Study
Source: PLoS One. 2021 Jan 28;16(1):e0245782. doi: 10.1371/journal.pone.0245782 (PMC7842951; doi:10.1371/journal.pone.0245782)
Supplement: S1 Table — (DOCX) [file pone.0245782.s001.docx]

| **S1 Table. Number of each quintile of vegetables and nutrients for the outcomes of interest** | | | | | | | | | | | | | |
| --- | --- | --- | --- | --- | --- | --- | --- | --- | --- | --- | --- | --- | --- |
| **Maternal intake^a^** | |  | **Asthma** |  | **Wheeze** |  | **Atopic dermatitis** |  | **Eczema** |  | **Food allergy** |  | **Any allergy** |
|  |  |  | Prevalence (%) |  | Prevalence (%) |  | Prevalence (%) |  | Prevalence (%) |  | Prevalence (%) |  | Prevalence (%) |
| **Total vegetables (g/day)** | | | |  |  |  |  |  |  |  |  |  |  |
|  | Q1 (55.7±33.8) |  | 350/13807 (2.53%) |  | 2701/13728 (19.68%) |  | 568/13807 (4.11%) |  | 2470/13740 (17.98%) |  | 2236/13807 (16.19%) |  | 4170/13751 (30.33%) |
|  | Q2 (94.9±37.5) |  | 367/14101 (2.60%) |  | 2741/14039 (19.52%) |  | 636/14101 (4.51%) |  | 2600/14050 (18.51%) |  | 2562/14101 (18.17%) |  | 4459/14063 (31.71%) |
|  | Q3 (125.5±47.0) |  | 346/14243 (2.43%) |  | 2728/14168 (19.25%) |  | 552/14243 (3.88%) |  | 2587/14178 (18.25%) |  | 2446/14243 (17.17%) |  | 4382/14189 (30.88%) |
|  | Q4 (160.7±67.7) |  | 343/14204 (2.41%) |  | 2752/14147 (19.45%) |  | 648/14204 (4.56%) |  | 2757/14137 (19.50%) |  | 2586/14204 (18.21%) |  | 4602/14148 (32.53%) |
|  | Q5 (264.2±184.7) |  | 345/13998 (2.46%) |  | 2742/13928 (19.69%) |  | 679/13998 (4.85%) |  | 2802/13939 (20.10%) |  | 2470/13998 (17.65%) |  | 4503/13952 (32.27%) |
| **Folate vegetables (g/day)** | | | |  |  |  |  |  |  |  |  |  |  |
|  | Q1 (9.0±6.4) |  | 404/13809 (2.93%) |  | 2771/13734 (20.18%) |  | 582/13809 (4.21%) |  | 2505/13736 (18.24%) |  | 2327/13809 (16.85%) |  | 4293/13753 (31.22%) |
|  | Q2 (24.1±16.1) |  | 355/14069 (2.52%) |  | 2707/13994 (19.34%) |  | 604/14069 (4.29%) |  | 2566/14020 (18.30%) |  | 2460/14069 (17.49%) |  | 4398/14026 (31.36%) |
|  | Q3 (27.5±10.5) |  | 337/14178 (2.38%) |  | 2787/14110 (19.75%) |  | 582/14178 (4.10%) |  | 2631/14116 (18.64%) |  | 2474/14178 (17.45%) |  | 4386/14128 (31.04%) |
|  | Q4 (38.4±14.9) |  | 352/14237 (2.47%) |  | 2773/14177 (19.56%) |  | 645/14237 (4.53%) |  | 2734/14171 (19.29%) |  | 2514/14237 (17.66%) |  | 4543/14180 (32.04%) |
|  | Q5 (69.0±54.1) |  | 303/14060 (2.16%) |  | 2626/13995 (18.76%) |  | 670/14060 (4.77%) |  | 2780/14001 (19.86%) |  | 2525/14060 (17.96%) |  | 4496/14016 (32.08%) |
| **Green and yellow vegetables (g/day)** | | | |  |  |  |  |  |  |  |  |  |  |
|  | Q1 (15.5±10.0) |  | 381/13773 (2.77%) |  | 2727/13700 (19.91%) |  | 569/13773 (4.13%) |  | 2441/13704 (17.81%) |  | 2199/13773 (15.97%) |  | 4144/13716 (30.21%) |
|  | Q2 (30.5±12.4) |  | 361/14068 (2.57%) |  | 2740/14001 (19.57%) |  | 599/14068 (4.26%) |  | 2552/14016 (18.21%) |  | 2404/14068 (17.09%) |  | 4287/14027 (30.56%) |
|  | Q3 (43.0±16.5) |  | 350/14162 (2.47%) |  | 2795/14097 (19.83%) |  | 610/14162 (4.31%) |  | 2626/14090 (18.64%) |  | 2501/14162 (17.66%) |  | 4477/14103 (31.75%) |
|  | Q4 (57.9±24.1) |  | 336/14264 (2.36%) |  | 2717/14183 (19.16%) |  | 642/14264 (4.50%) |  | 2796/14199 (19.69%) |  | 2614/14264 (18.33%) |  | 4619/14207 (32.51%) |
|  | Q5 (102.1±73.4) |  | 323/14086 (2.29%) |  | 2685/14029 (19.14%) |  | 663/14086 (4.71%) |  | 2801/14035 (19.96%) |  | 2582/14086 (18.33%) |  | 4589/14050 (32.66%) |
| **Cruciferous vegetables (g/day)** | | | |  |  |  |  |  |  |  |  |  |  |
|  | Q1 (10.3±6.6) |  | 388/13874 (2.80%) |  | 2698/13802 (19.55%) |  | 607/13874 (4.38%) |  | 2542/13810 (18.41%) |  | 2425/13874 (17.48%) |  | 4383/13827 (31.70%) |
|  | Q2 (21.1±8.0) |  | 322/14115 (2.28%) |  | 2772/14046 (19.74%) |  | 584/14115 (4.14%) |  | 2624/14052 (18.67%) |  | 2513/14115 (17.80%) |  | 4440/14065 (31.57%) |
|  | Q3 (30.4±11.6) |  | 365/14194 (2.57%) |  | 2771/14138 (19.60%) |  | 627/14194 (4.42%) |  | 2644/14136 (18.70%) |  | 2510/14194 (17.68%) |  | 4448/14147 (31.44%) |
|  | Q4 (42.4±15.0) |  | 360/14151 (2.54%) |  | 2732/14074 (19.41%) |  | 637/14151 (4.50%) |  | 2680/14093 (19.02%) |  | 2460/14151 (17.38%) |  | 4450/14102 (31.56%) |
|  | Q5 (75.2±56.4) |  | 316/14019 (2.25%) |  | 2691/13950 (19.29%) |  | 628/14019 (4.48%) |  | 2726/13953 (19.54%) |  | 2392/14019 (17.06%) |  | 4395/13962 (31.48%) |
| **Vitamin A (µg/day)** | |  |  |  |  |  |  |  |  |  |  |  |  |
|  | Q1 (240.5±19.3) |  | 421/13857 (3.04%) |  | 2850/13796 (20.66%) |  | 640/13857 (4.62%) |  | 2502/13800 (18.13%) |  | 2217/13857 (16.00%) |  | 4179/13812 (30.26%) |
|  | Q2 (353.0±187.4) |  | 344/14127 (2.44%) |  | 2680/14051 (19.07%) |  | 595/14127 (4.21%) |  | 2601/14058 (18.50%) |  | 2525/14127 (17.87%) |  | 4454/14073 (31.65%) |
|  | Q3 (449.7±247.1) |  | 324/14186 (2.28%) |  | 2678/14115 (18.97%) |  | 612/14186 (4.31%) |  | 2731/14128 (19.33%) |  | 2570/14186 (18.12%) |  | 4560/14138 (32.25%) |
|  | Q4 (584.4±330.6) |  | 325/14152 (2.30%) |  | 2688/14091 (19.08%) |  | 610/14152 (4.31%) |  | 2715/14090 (19.27%) |  | 2589/14152 (18.29%) |  | 4557/14105 (32.31%) |
|  | Q5 (1053.6±1265.4) | | 337/14031 (2.40%) |  | 2768/13957 (19.83%) |  | 626/14031 (4.46%) |  | 2667/13968 (19.09%) |  | 2399/14031 (17.10%) |  | 4366/13975 (31.24%) |
| **Alpha-carotene (µg/day)** | | | |  |  |  |  |  |  |  |  |  |  |
|  | Q1 (102.8±81.4) |  | 339/13786 (2.46%) |  | 2611/13709 (19.05%) |  | 595/13786 (4.32%) |  | 2475/13717 (18.04%) |  | 2246/13786 (16.29%) |  | 4149/13730 (30.22%) |
|  | Q2 (255.7±106.7) |  | 349/14071 (2.48%) |  | 2669/14014 (19.05%) |  | 613/14071 (4.36%) |  | 2646/14009 (18.89%) |  | 2460/14071 (17.48%) |  | 4426/14020 (31.57%) |
|  | Q3 (377.4±190.7) |  | 342/14139 (2.42%) |  | 2795/14066 (19.87%) |  | 592/14139 (4.19%) |  | 2564/14084 (18.21%) |  | 2516/14139 (17.79%) |  | 4382/14097 (31.08%) |
|  | Q4 (581.8±215.6) |  | 381/14182 (2.69%) |  | 2832/14109 (20.07%) |  | 628/14182 (4.43%) |  | 2754/14118 (19.51%) |  | 2511/14182 (17.71%) |  | 4593/14126 (32.51%) |
|  | Q5 (1061.2±948.3) | | 340/14175 (2.40%) |  | 2757/14112 (19.54%) |  | 655/14175 (4.62%) |  | 2777/14116 (19.67%) |  | 2567/14175 (18.11%) |  | 4566/14130 (32.31%) |
| **Beta-carotene (µg)/day** | | | |  |  |  |  |  |  |  |  |  |  |
|  | Q1 (1127.6±771.4) | | 378/13794 (2.74%) |  | 2686/13724 (19.57%) |  | 596/13794 (4.32%) |  | 2483/13723 (18.09%) |  | 2247/13794 (16.29%) |  | 4185/13733 (30.47%) |
|  | Q2 (1966.5±895.1) | | 325/14082 (2.31%) |  | 2706/14018 (19.30%) |  | 575/14082 (4.08%) |  | 2573/14034 (18.33%) |  | 2455/14082 (17.43%) |  | 4334/14049 (30.85%) |
|  | Q3 (2678.3±1247.3) | | 367/14192 (2.59%) |  | 2807/14116 (19.89%) |  | 625/14192 (4.40%) |  | 2632/14124 (18.63%) |  | 2535/14192 (17.86%) |  | 4497/14133 (31.82%) |
|  | Q4 (3508.8±1398.7) | | 344/14149 (2.43%) |  | 2731/14076 (19.40%) |  | 629/14149 (4.45%) |  | 2761/14078 (19.61%) |  | 2542/14149 (17.97%) |  | 4571/14093 (32.43%) |
|  | Q5 (5753.5±4278.8) | | 337/14136 (2.38%) |  | 2734/14076 (19.42%) |  | 658/14136 (4.65%) |  | 2767/14085 (19.65%) |  | 2521/14136 (17.83%) |  | 4529/14095 (32.13%) |
| **Vitamin C (mg/day)** | | |  |  |  |  |  |  |  |  |  |  |  |
|  | Q1 (39.0±25.2) |  | 371/13893 (2.67%) |  | 2714/13821 (19.64%) |  | 587/13893 (4.23%) |  | 2471/13832 (17.86%) |  | 2295/13893 (16.52%) |  | 4198/13839 (30.33%) |
|  | Q2 (61.5±26.8) |  | 349/14119 (2.47%) |  | 2780/14068 (19.76%) |  | 585/14119 (4.14%) |  | 2706/14064 (19.24%) |  | 2514/14119 (17.81%) |  | 4497/14078 (31.94%) |
|  | Q3 (81.5±41.3) |  | 346/14184 (2.44%) |  | 2775/14119 (19.65%) |  | 617/14184 (4.35%) |  | 2634/14112 (18.66%) |  | 2545/14184 (17.94%) |  | 4492/14130 (31.79%) |
|  | Q4 (102.9±47.2) |  | 351/14133 (2.48%) |  | 2674/14052 (19.03%) |  | 644/14133 (4.56%) |  | 2698/14089 (19.15%) |  | 2531/14133 (17.91%) |  | 4506/14094 (31.97%) |
|  | Q5 (157.1±98.3) |  | 334/14024 (2.38%) |  | 2721/13950 (19.51%) |  | 650/14024 (4.63%) |  | 2707/13947 (19.41%) |  | 2415/14024 (17.22%) |  | 4423/13962 (31.68%) |
| **Alpha-tocopherol (mg/day)** | | | |  |  |  |  |  |  |  |  |  |  |
|  | Q1 (4.2±2.3) |  | 404/13806 (2.93%) |  | 2831/13735 (20.61%) |  | 576/13806 (4.17%) |  | 2498/13744 (18.18%) |  | 2232/13806 (16.17%) |  | 4214/13754 (30.64%) |
|  | Q2 5.4±2.3) |  | 373/14097 (2.65%) |  | 2769/14032 (19.73%) |  | 609/14097 (4.32%) |  | 2594/14041 (18.47%) |  | 2397/14097 (17.00%) |  | 4355/14050 (31.00%) |
|  | Q3 (6.2±2.5) |  | 370/14224 (2.60%) |  | 2749/14157 (19.42%) |  | 642/14224 (4.51%) |  | 2666/14161 (18.83%) |  | 2512/14224 (17.66%) |  | 4522/14177 (31.90%) |
|  | Q4 (7.1±3.2) |  | 306/14082 (2.17%) |  | 2666/14011 (19.03%) |  | 616/14082 (4.37%) |  | 2721/14025 (19.40%) |  | 2576/14082 (18.29%) |  | 4535/14035 (32.31%) |
|  | Q5 (9.3±5.5) |  | 298/14144 (2.11%) |  | 2649/14075 (18.82%) |  | 640/14144 (4.52%) |  | 2737/14073 (19.45%) |  | 2583/14144 (18.26%) |  | 4490/14087 (31.87%) |
| **Vitamin K (µg/day)** | |  |  |  |  |  |  |  |  |  |  |  |  |
|  | Q1 (82.2±50.4) |  | 351/13966 (2.51%) |  | 2699/13894 (19.43%) |  | 607/13966 (4.35%) |  | 2542/13910 (18.27%) |  | 2423/13966 (17.35%) |  | 4348/13921 (31.23%) |
|  | Q2 (129.7±60.9) |  | 368/14160 (2.60%) |  | 2801/14089 (19.88%) |  | 613/14160 (4.33%) |  | 2676/14105 (18.97%) |  | 2543/14160 (17.96%) |  | 4524/14116 (32.05%) |
|  | Q3 (167.3±75.3) |  | 368/14158 (2.60%) |  | 2758/14093 (19.57%) |  | 599/14158 (4.23%) |  | 2646/14087 (18.78%) |  | 2462/14158 (17.39%) |  | 4456/14097 (31.61%) |
|  | Q4 (221.8±105.1) |  | 336/14065 (2.39%) |  | 2769/13997 (19.78%) |  | 671/14065 (4.77%) |  | 2683/13999 (19.17%) |  | 2495/14065 (17.74%) |  | 4449/14012 (31.75%) |
|  | Q5 (358.9±255.6) |  | 328/14004 (2.34%) |  | 2637/13937 (18.92%) |  | 593/14004 (4.23%) |  | 2669/13943 (19.14%) |  | 2377/14004 (16.97%) |  | 4339/13957 (31.09%) |
| **Folate (µg/day)** | |  |  |  |  |  |  |  |  |  |  |  |  |
|  | Q1 (158.2±873.9) |  | 397/13846 (2.87%) |  | 2740/13785 (19.88%) |  | 606/13846 (4.38%) |  | 2451/13789 (17.78%) |  | 2364/13846 (17.07%) |  | 4263/13802 (30.89%) |
|  | Q2 (210.6±92.4) |  | 328/14133 (2.32%) |  | 2744/14070 (19.50%) |  | 577/14133 (4.08%) |  | 2674/14078 (18.99%) |  | 2515/14133 (17.80%) |  | 4480/14085 (31.81%) |
|  | Q3 (247.8±100.4) |  | 348/14201 (2.45%) |  | 2719/14123 (19.25%) |  | 617/14201 (4.34%) |  | 2714/14142 (19.19%) |  | 2520/14201 (17.75%) |  | 4506/14160 (31.82%) |
|  | Q4 (293.8±134.1) |  | 351/14194 (2.47%) |  | 2722/14138 (19.25%) |  | 648/14194 (4.57%) |  | 2692/14135 (19.04%) |  | 2486/14194 (17.51%) |  | 4500/14141 (31.82%) |
|  | Q5 (394.6±230.0) |  | 327/13979 (2.34%) |  | 2739/13894 (19.71%) |  | 635/13979 (4.54%) |  | 2685/13900 (19.32%) |  | 2415/13979 (17.28%) |  | 4367/13915 (31.38%) |
| **Soluble fiber (g/day)** | | |  |  |  |  |  |  |  |  |  |  |  |
|  | Q1 (1.5±0.7) |  | 380/13839 (2.75%) |  | 2753/13763 (20.00%) |  | 573/13839 (4.14%) |  | 2403/13775 (17.44%) |  | 2360/13839 (17.05%) |  | 4208/13786 (30.52%) |
|  | Q2 (2.0±0.7) |  | 355/14119 (2.51%) |  | 2776/14065 (19.74%) |  | 585/14119 (4.14%) |  | 2563/14060 (18.23%) |  | 2538/14119 (17.98%) |  | 4455/14068 (31.67%) |
|  | Q3 (2.4±0.9) |  | 337/14243 (2.37%) |  | 2715/14163 (19.17%) |  | 643/14243 (4.51%) |  | 2729/14187 (19.24%) |  | 2367/14243 (16.62%) |  | 4389/14199 (30.91%) |
|  | Q4 (2.9±1.0) |  | 347/14108 (2.46%) |  | 2753/14046 (19.60%) |  | 643/14108 (4.56%) |  | 2710/14045 (19.30%) |  | 2525/14108 (17.90%) |  | 4513/14061 (32.10%) |
|  | Q5 (4.3±2.6) |  | 332/14044 (2.36%) |  | 2667/13973 (19.09%) |  | 639/14044 (4.55%) |  | 2811/13977 (20.11%) |  | 2510/14044 (17.87%) |  | 4551/13989 (32.53%) |
| **Insoluble fiber (g/day)** | | | |  |  |  |  |  |  |  |  |  |  |
|  | Q1 (5.0±2.3) |  | 380/13784 (2.76%) |  | 2774/13706 (20.24%) |  | 585/13784 (4.24%) |  | 2404/13720 (17.52%) |  | 2377/13784 (17.24%) |  | 4212/13729 (30.68%) |
|  | Q2 (6.2±2.3) |  | 349/14151 (2.47%) |  | 2710/14104 (19.21%) |  | 604/14151 (4.27%) |  | 2618/14089 (18.58%) |  | 2502/14151 (17.68%) |  | 4444/14100 (31.52%) |
|  | Q3 (7.3±2.6) |  | 355/14204 (2.50%) |  | 2749/14122 (19.47%) |  | 598/14204 (4.21%) |  | 2667/14144 (18.86%) |  | 2464/14204 (17.35%) |  | 4464/14158 (31.53%) |
|  | Q4 (8.6±3.2) |  | 340/14196 (2.40%) |  | 2719/14127 (19.25%) |  | 652/14196 (4.59%) |  | 2757/14139 (19.50%) |  | 2486/14196 (17.51%) |  | 4526/14154 (31.98%) |
|  | Q5 (11.8±6.8) |  | 327/14018 (2.33%) |  | 2712/13951 (19.44%) |  | 644/14018 (4.59%) |  | 2770/13952 (19.85%) |  | 2471/14018 (17.63%) |  | 4470/13962 (32.02%) |
| **Total fiber (g/day)** | |  |  |  |  |  |  |  |  |  |  |  |  |
|  | Q1 (6.8±3.2) |  | 375/13800 (2.72%) |  | 2742/13730 (19.97%) |  | 578/13800 (4.19%) |  | 2404/13736 (17.50%) |  | 2362/13800 (17.12%) |  | 4189/13745 (30.48%) |
|  | Q2 (8.7±3.3) |  | 366/14119 (2.59%) |  | 2768/14059 (19.69%) |  | 613/14119 (4.34%) |  | 2630/14054 (18.71%) |  | 2520/14119 (17.85%) |  | 4483/14067 (31.87%) |
|  | Q3 (10..3±3.9) |  | 339/14241 (2.38%) |  | 2761/14165 (19.49%) |  | 608/14241 (4.27%) |  | 2664/14183 (18.78%) |  | 2415/14241 (16.96%) |  | 4426/14195 (31.18%) |
|  | Q4 (12.0±4.5) |  | 352/14153 (2.49%) |  | 2687/14084 (19.08%) |  | 645/14153 (4.56%) |  | 2725/14098 (19.33%) |  | 2510/14153 (17.73%) |  | 4509/14111 (31.95%) |
|  | Q5 (16.4±9.6) |  | 319/14040 (2.27%) |  | 2706/13972 (19.37%) |  | 639/14040 (4.55%) |  | 2793/13973 (19.99%) |  | 2493/14040 (17.76%) |  | 4509/13985 (32.24%) |
| a: Q1 is the lowest quintile. Q5 is the highest quintile. | | | | | |  |  |  |  |  |  |  |  |
