# Supplementary material for: Association between maternal vegetable intake during pregnancy and allergy in offspring: Japan Environment and Children’s Study
Source: PLoS One. 2021 Jan 28;16(1):e0245782. doi: 10.1371/journal.pone.0245782 (PMC7842951; doi:10.1371/journal.pone.0245782)
Supplement: S2 Table — (DOCX) [file pone.0245782.s002.docx]

| **S2 Table. Crude odds ratio of the allergic outcomes between quintiles for each exposure category** | | | | | | | | | | | | | |
| --- | --- | --- | --- | --- | --- | --- | --- | --- | --- | --- | --- | --- | --- |
| **Maternal intake^a^** | |  | **Asthma** |  | **Wheeze** |  | **Atopic dermatitis** | | **Eczema** |  | **Food allergy** |  | **Any allergy** |
|  |  |  | OR |  | OR |  | OR |  | OR |  | OR |  | OR |
| **Total vegetables (g/day)** | | | |  |  |  |  |  |  |  |  |  |  |
|  | Q1 (55.7±33.8) | | Reference |  | Reference |  | Reference |  | Reference |  | Reference |  | Reference |
|  | Q2 (94.9±37.5) | | 1.00 (0.87-1.15) |  | 0.99 (0.94-1.05) |  | 1.07 (0.96-1.19) |  | 1.04 (0.98-1.10) |  | 1.14 (1.07-1.20) |  | 1.06 (1.01-1.11) |
|  | Q3 (125.5±47.0) | | 0.95 (0.83-1.09) |  | 0.98 (0.92-1.03) |  | 0.94 (0.84-1.05) |  | 1.01 (0.96-1.07) |  | 1.07 (1.01-1.13) |  | 1.02 (0.97-1.07) |
|  | Q4 (160.7±67.7) | | 0.93 (0.81-1.07) |  | 0.97 (0.92-1.02) |  | 1.09 (0.97-1.21) |  | 1.08 (1.02-1.14) |  | 1.14 (1.07-1.21) |  | 1.08 (1.03-1.14) |
|  | Q5 (264.2±184.7) | | 0.96 (0.83-1.10) |  | 1.00 (0.95-1.06) |  | 1.15 (1.03-1.28) |  | 1.14 (1.07-1.20) |  | 1.10 (1.04-1.17) |  | 1.08 (1.03-1.13) |
| **Folate vegetables (g/day)** | | | |  |  |  |  |  |  |  |  |  |  |
|  | Q1 (9.0±6.4) |  | Reference |  | Reference |  | Reference |  | Reference |  | Reference |  | Reference |
|  | Q2 (24.1±16.1) | | 0.85 (0.75-0.98) |  | 0.96 (0.91-1.01) |  | 0.97 (0.87-1.09) |  | 0.98 (0.93-1.04) |  | 1.03 (0.98-1.10) |  | 0.99 (0.94-1.03) |
|  | Q3 (27.5±10.5) | | 0.80 (0.70-0.92) |  | 0.96 (0.91-1.02) |  | 0.97 (0.87-1.08) |  | 1.00 (0.94-1.05) |  | 1.04 (0.98-1.10) |  | 0.97 (0.93-1.02) |
|  | Q4 (38.4±14.9) | | 0.83 (0.73-0.95) |  | 0.95 (0.90-1.01) |  | 1.05 (0.94-1.17) |  | 1.04 (0.98-1.10) |  | 1.04 (0.98-1.10) |  | 1.01 (0.96-1.06) |
|  | Q5 (69.0±54.1) | | 0.73 (0.63-0.84) |  | 0.91 (0.86-0.96) |  | 1.11 (1.00-1.23) |  | 1.09 (1.03-1.15) |  | 1.07 (1.01-1.13) |  | 1.02 (0.97-1.07) |
| **Green and yellow vegetables (g/day)** | | | | | |  |  |  |  |  |  |  |  |
|  | Q1 (15.5±10.0) | | Reference |  | Reference |  | Reference |  | Reference |  | Reference |  | Reference |
|  | Q2 (30.5±12.4) | | 0.87 (0.76-1.00) |  | 0.97 (0.92-1.02) |  | 1.02 (0.91-1.14) |  | 1.02 (0.97-1.08) |  | 1.08 (1.02-1.15) |  | 1.01 (0.96-1.06) |
|  | Q3 (43.0±16.5) | | 0.88 (0.77-1.01) |  | 0.98 (0.93-1.03) |  | 1.03 (0.93-1.15) |  | 1.05 (0.99-1.11) |  | 1.12 (1.06-1.19) |  | 1.06 (1.01-1.11) |
|  | Q4 (57.9±24.1) | | 0.84 (0.73-0.96) |  | 0.93 (0.88-0.98) |  | 1.05 (0.95-1.17) |  | 1.11 (1.05-1.18) |  | 1.17 (1.10-1.24) |  | 1.10 (1.05-1.15) |
|  | Q5 (102.1±73.4) | | 0.79 (0.69-0.91) |  | 0.94 (0.89-0.99) |  | 1.12 (1.01-1.25) |  | 1.14 (1.08-1.21) |  | 1.17 (1.10-1.24) |  | 1.10 (1.05-1.16) |
| **Cruciferous vegetables (g/day)** | | | |  |  |  |  |  |  |  |  |  |  |
|  | Q1 (10.3±6.6) |  | Reference |  | Reference |  | Reference |  | Reference |  | Reference |  | Reference |
|  | Q2 (21.1±8.0) |  | 0.83 (0.72-0.95) |  | 0.99 (0.94-1.04) |  | 0.94 (0.85-1.05) |  | 1.01 (0.96-1.07) |  | 1.01 (0.95-1.07) |  | 0.99 (0.94-1.03) |
|  | Q3 (30.4±11.6) | | 0.89 (0.78-1.02) |  | 0.99 (0.93-1.04) |  | 0.99 (0.89-1.10) |  | 1.02 (0.97-1.08) |  | 1.02 (0.96-1.08) |  | 0.98 (0.94-1.03) |
|  | Q4 (42.4±15.0) | | 0.93 (0.81-1.06) |  | 0.99 (0.94-1.05) |  | 1.02 (0.92-1.13) |  | 1.04 (0.98-1.10) |  | 0.99 (0.94-1.05) |  | 0.99 (0.94-1.04) |
|  | Q5 (75.2±56.4) | | 0.80 (0.70-0.92) |  | 0.97 (0.92-1.02) |  | 0.99 (0.89-1.10) |  | 1.07 (1.01-1.13) |  | 0.96 (0.90-1.01) |  | 0.98 (0.93-1.03) |
| **Vitamin A (µg/day)** | | | |  |  |  |  |  |  |  |  |  |  |
|  | Q1 (240.5±19.3) | | Reference |  | Reference |  | Reference |  | Reference |  | Reference |  | Reference |
|  | Q2 (353.0±187.4) | | 0.77 (0.67-0.88) |  | 0.89 (0.84-0.94) |  | 0.92 (0.82-1.02) |  | 1.01 (0.96-1.07) |  | 1.13 (1.06-1.20) |  | 1.05 (1.00-1.10) |
|  | Q3 (449.7±247.1) | | 0.76 (0.66-0.87) |  | 0.88 (0.83-0.93) |  | 0.93 (0.84-1.04) |  | 1.06 (1.00-1.12) |  | 1.15 (1.09-1.22) |  | 1.08 (1.03-1.14) |
|  | Q4 (584.4±330.6) | | 0.75 (0.65-0.85) |  | 0.89 (0.85-0.94) |  | 0.91 (0.82-1.02) |  | 1.06 (1.01-1.13) |  | 1.18 (1.11-1.25) |  | 1.09 (1.04-1.15) |
|  | Q5 (1053.6±1265.4) | | 0.77 (0.67-0.88) |  | 0.93 (0.88-0.98) |  | 0.95 (0.86-1.06) |  | 1.05 (0.99-1.11) |  | 1.08 (1.01-1.14) |  | 1.03 (0.98-1.08) |
| **Alpha-carotene (µg/day)** | | | |  |  |  |  |  |  |  |  |  |  |
|  | Q1 (102.8±81.4) | | Reference |  | Reference |  | Reference |  | Reference |  | Reference |  | Reference |
|  | Q2 (255.7±106.7) | | 0.96 (0.84-1.10) |  | 0.98 (0.93-1.04) |  | 1.02 (0.91-1.13) |  | 1.06 (1.00-1.12) |  | 1.08 (1.02-1.14) |  | 1.06 (1.01-1.11) |
|  | Q3 (377.4±190.7) | | 0.98 (0.85-1.12) |  | 1.03 (0.97-1.09) |  | 0.99 (0.89-1.10) |  | 1.01 (0.95-1.07) |  | 1.10 (1.04-1.17) |  | 1.04 (0.99-1.09) |
|  | Q4 (581.8±215.6) | | 1.07 (0.93-1.23) |  | 1.03 (0.98-1.09) |  | 1.00 (0.90-1.12) |  | 1.09 (1.03-1.15) |  | 1.09 (1.02-1.15) |  | 1.09 (1.04-1.15) |
|  | Q5 (1061.2±948.3) | | 0.94 (0.81-1.08) |  | 1.01 (0.96-1.07) |  | 1.05 (0.95-1.17) |  | 1.10 (1.04-1.16) |  | 1.12 (1.05-1.18) |  | 1.09 (1.04-1.14) |
| **Beta-carotene (µg/day)** | | | |  |  |  |  |  |  |  |  |  |  |
|  | Q1 (1127.6±771.4) | | Reference |  | Reference |  | Reference |  | Reference |  | Reference |  | Reference |
|  | Q2 (1966.5±895.1) | | 0.82 (0.72-0.94) |  | 0.97 (0.92-1.02) |  | 0.94 (0.84-1.05) |  | 1.01 (0.96-1.07) |  | 1.08 (1.02-1.15) |  | 1.01 (0.96-1.06) |
|  | Q3 (2678.3±1247.3) | | 0.91 (0.79-1.04) |  | 1.00 (0.95-1.05) |  | 1.02 (0.91-1.13) |  | 1.03 (0.98-1.09) |  | 1.10 (1.04-1.17) |  | 1.05 (1.00-1.10) |
|  | Q4 (3508.8±1398.7) | | 0.87 (0.77-1.01) |  | 0.96 (0.91-1.01) |  | 0.99 (0.89-1.10) |  | 1.07 (1.02-1.14) |  | 1.12 (1.05-1.18) |  | 1.07 (1.02-1.12) |
|  | Q5 (5753.5±4278.8) | | 0.83 (0.73-0.96) |  | 0.97 (0.92-1.02) |  | 1.05 (0.95-1.17) |  | 1.10 (1.04-1.16) |  | 1.10 (1.04-1.17) |  | 1.07 (1.02-1.12) |
| **Vitamin C (mg/day)** | | | |  |  |  |  |  |  |  |  |  |  |
|  | Q1 (39.0±25.2) | | Reference |  | Reference |  | Reference |  | Reference |  | Reference |  | Reference |
|  | Q2 (61.5±26.8) | | 0.90 (0.79-1.03) |  | 1.00 (0.95-1.06) |  | 0.98 (0.88-1.10) |  | 1.08 (1.02-1.15) |  | 1.09 (1.03-1.15) |  | 1.06 (1.02-1.12) |
|  | Q3 (81.5±41.3) | | 0.88 (0.77-1.01) |  | 0.99 (0.93-1.04) |  | 1.03 (0.93-1.15) |  | 1.05 (0.99-1.11) |  | 1.11 (1.05-1.18) |  | 1.06 (1.01-1.11) |
|  | Q4 (102.9±47.2) | | 0.88 (0.77-1.01) |  | 0.96 (0.91-1.02) |  | 1.06 (0.95-1.18) |  | 1.07 (1.01-1.13) |  | 1.10 (1.04-1.17) |  | 1.07 (1.02-1.12) |
|  | Q5 (157.1±98.3) | | 0.83 (0.72-0.96) |  | 0.98 (0.93-1.04) |  | 1.07 (0.96-1.19) |  | 1.09 (1.03-1.16) |  | 1.06 (1.00-1.12) |  | 1.05 (1.00-1.10) |
| **Alpha-tocopherol (mg/day)** | | | |  |  |  |  |  |  |  |  |  |  |
|  | Q1 (4.2±2.3) |  | Reference |  | Reference |  | Reference |  | Reference |  | Reference |  | Reference |
|  | Q2 5.4±2.3) |  | 0.88 (0.77-1.01) |  | 0.94 (0.89-1.00) |  | 1.04 (0.93-1.16) |  | 1.01 (0.96-1.07) |  | 1.05 (0.99-1.11) |  | 1.00 (0.95-1.05) |
|  | Q3 (6.2±2.5) |  | 0.87 (0.76-1.00) |  | 0.93 (0.88-0.98) |  | 1.07 (0.96-1.20) |  | 1.03 (0.97-1.09) |  | 1.09 (1.03-1.16) |  | 1.03 (0.99-1.08) |
|  | Q4 (7.1±3.2) |  | 0.76 (0.66-0.87) |  | 0.91 (0.86-0.96) |  | 1.08 (0.97-1.20) |  | 1.08 (1.02-1.15) |  | 1.17 (1.10-1.24) |  | 1.08 (1.03-1.13) |
|  | Q5 (9.3±5.5) |  | 0.72 (0.63-0.83) |  | 0.89 (0.84-0.94) |  | 1.06 (0.95-1.18) |  | 1.08 (1.02-1.14) |  | 1.14 (1.08-1.21) |  | 1.04 (0.99-1.09) |
| **Vitamin K (µg/day)** | | | |  |  |  |  |  |  |  |  |  |  |
|  | Q1 (82.2±50.4) | | Reference |  | Reference |  | Reference |  | Reference |  | Reference |  | Reference |
|  | Q2 (129.7±60.9) | | 1.03 (0.90-1.18) |  | 1.03 (0.97-1.09) |  | 1.02 (0.92-1.14) |  | 1.05 (0.99-1.11) |  | 1.05 (0.99-1.11) |  | 1.04 (0.99-1.09) |
|  | Q3 (167.3±75.3) | | 1.03 (0.90-1.19) |  | 1.01 (0.96-1.07) |  | 0.99 (0.89-1.10) |  | 1.03 (0.97-1.09) |  | 1.02 (0.96-1.08) |  | 1.02 (0.97-1.07) |
|  | Q4 (221.8±105.1) | | 0.95 (0.83-1.09) |  | 1.02 (0.96-1.08) |  | 1.11 (1.00-1.23) |  | 1.06 (1.00-1.12) |  | 1.03 (0.97-1.09) |  | 1.02 (0.98-1.07) |
|  | Q5 (358.9±255.6) | | 0.93 (0.81-1.07) |  | 0.97 (0.92-1.03) |  | 0.97 (0.87-1.08) |  | 1.05 (0.99-1.11) |  | 0.98 (0.92-1.04) |  | 0.99 (0.95-1.04) |
| **Folate (µg/day)** | | | |  |  |  |  |  |  |  |  |  |  |
|  | Q1 (158.2±873.9) | | Reference |  | Reference |  | Reference |  | Reference |  | Reference |  | Reference |
|  | Q2 (210.6±92.4) | | 0.80 (0.69-0.91) |  | 0.96 (0.91-1.01) |  | 0.93 (0.84-1.04) |  | 1.06 (1.00-1.12) |  | 1.04 (0.98-1.10) |  | 1.03 (0.98-1.07) |
|  | Q3 (247.8±100.4) | | 0.88 (0.77-1.00) |  | 0.95 (0.90-1.01) |  | 1.01 (0.91-1.12) |  | 1.10 (1.03-1.16) |  | 1.05 (0.99-1.11) |  | 1.05 (1.00-1.10) |
|  | Q4 (293.8±134.1) | | 0.83 (0.72-0.95) |  | 0.95 (0.90-1.00) |  | 1.05 (0.94-1.17) |  | 1.07 (1.01-1.14) |  | 1.03 (0.97-1.09) |  | 1.03 (0.98-1.08) |
|  | Q5 (394.6±230.0) | | 0.80 (0.70-0.92) |  | 0.98 (0.93-1.03) |  | 1.02 (0.91-1.13) |  | 1.09 (1.03-1.15) |  | 1.01 (0.95-1.07) |  | 1.01 (0.96-1.06) |
| **Soluble fiber (g/day)** | | | |  |  |  |  |  |  |  |  |  |  |
|  | Q1 (1.5±0.7) |  | Reference |  | Reference |  | Reference |  | Reference |  | Reference |  | Reference |
|  | Q2 (2.0±0.7) |  | 0.92 (0.80-1.05) |  | 1.00 (0.95-1.06) |  | 1.01 (0.91-1.13) |  | 1.04 (0.98-1.10) |  | 1.07 (1.01-1.14) |  | 1.05 (1.00-1.10) |
|  | Q3 (2.4±0.9) |  | 0.85 (0.74-0.98) |  | 0.94 (0.89-0.99) |  | 1.08 (0.97-1.21) |  | 1.10 (1.04-1.17) |  | 0.99 (0.93-1.05) |  | 1.02 (0.97-1.07) |
|  | Q4 (2.9±1.0) |  | 0.87 (0.76-1.00) |  | 0.97 (0.92-1.03) |  | 1.12 (1.00-1.24) |  | 1.13 (1.06-1.19) |  | 1.06 (1.00-1.13) |  | 1.07 (1.02-1.12) |
|  | Q5 (4.3±2.6) |  | 0.86 (0.75-0.99) |  | 0.95 (0.90-1.00) |  | 1.09 (0.98-1.22) |  | 1.16 (1.10-1.23) |  | 1.07 (1.01-1.13) |  | 1.09 (1.04-1.14) |
| **Insoluble fiber (g/day)** | | | |  |  |  |  |  |  |  |  |  |  |
|  | Q1 (5.0±2.3) |  | Reference |  | Reference |  | Reference |  | Reference |  | Reference |  | Reference |
|  | Q2 (6.2±2.3) |  | 0.90 (0.79-1.03) |  | 0.94 (0.89-0.99) |  | 1.01 (0.90-1.12) |  | 1.07 (1.01-1.14) |  | 1.03 (0.97-1.09) |  | 1.05 (1.00-1.10) |
|  | Q3 (7.3±2.6) |  | 0.88 (0.77-1.01) |  | 0.95 (0.90-1.01) |  | 0.97 (0.87-1.09) |  | 1.08 (1.02-1.15) |  | 1.01 (0.95-1.07) |  | 1.03 (0.99-1.08) |
|  | Q4 (8.6±3.2) |  | 0.87 (0.76-1.00) |  | 0.92 (0.87-0.97) |  | 1.10 (0.99-1.22) |  | 1.14 (1.07-1.20) |  | 1.02 (0.97-1.09) |  | 1.06 (1.01-1.11) |
|  | Q5 (11.8±6.8) |  | 0.84 (0.73-0.96) |  | 0.95 (0.90-1.00) |  | 1.08 (0.97-1.20) |  | 1.14 (1.08-1.21) |  | 1.03 (0.97-1.09) |  | 1.06 (1.01-1.11) |
| **Total fiber (g/day)** | | | |  |  |  |  |  |  |  |  |  |  |
|  | Q1 (6.8±3.2) |  | Reference |  | Reference |  | Reference |  | Reference |  | Reference |  | Reference |
|  | Q2 (8.7±3.3) |  | 0.93 (0.81-1.06) |  | 0.98 (0.93-1.03) |  | 1.03 (0.92-1.15) |  | 1.07 (1.01-1.13) |  | 1.05 (0.99-1.11) |  | 1.05 (1.00-1.10) |
|  | Q3 (10..3±3.9) | | 0.86 (0.75-0.99) |  | 0.96 (0.91-1.02) |  | 1.00 (0.90-1.12) |  | 1.06 (1.01-1.13) |  | 0.99 (0.93-1.05) |  | 1.02 (0.97-1.07) |
|  | Q4 (12.0±4.5) |  | 0.89 (0.77-1.02) |  | 0.93 (0.88-0.98) |  | 1.09 (0.98-1.22) |  | 1.12 (1.06-1.18) |  | 1.04 (0.98-1.10) |  | 1.06 (1.01-1.11) |
|  | Q5 (16.4±9.6) |  | 0.83 (0.72-0.95) |  | 0.96 (0.91-1.01) |  | 1.07 (0.96-1.20) |  | 1.15 (1.09-1.21) |  | 1.05 (0.99-1.11) |  | 1.07 (1.02-1.12) |
| a: Q1 is the lowest quintile. Q5 is the highest quintile | | | | | |  |  |  |  |  |  |  |  |
