# Supplementary material for: Association between maternal vegetable intake during pregnancy and allergy in offspring: Japan Environment and Children’s Study
Source: PLoS One. 2021 Jan 28;16(1):e0245782. doi: 10.1371/journal.pone.0245782 (PMC7842951; doi:10.1371/journal.pone.0245782)
Supplement: S4 Table — (DOCX) [file pone.0245782.s004.docx]

| **S4 Table. Association between maternal vegetable intake and maternal allergic history** | | | | | | | | |
| --- | --- | --- | --- | --- | --- | --- | --- | --- |
| **Total Vegetable Category** | **Maternal Allergy History** | **Asthma History** | **Allergic Rhinitis History** | **Atopic Dermatitis History** | **Conjunctivitis History** | **Food Allergy History** | **Drug Allergy History** | **Contact Dermatitis History** |
| Q1 | 7648 (47.9%) | 1748 (10.9%) | 5309 (33.2%) | 2366 (14.8%) | 1383 (8.7%) | 700 (4.4%) | 329 (2.1%) | 244 (1.5%) |
| Q2 | 8210 (51.3%) | 1725 (10.8%) | 5847 (36.5%) | 2557 (16.0%) | 1545 (9.7%) | 707 (4.4%) | 389 (2.4%) | 284 (1.8%) |
| Q3 | 8310 (52.0%) | 1708 (10.7%) | 5961 (37.3%) | 2577 (16.1%) | 1632 (10.2%) | 744 (4.7%) | 423 (2.6%) | 328 (2.1%) |
| Q4 | 8285 (51.8%) | 1699 (10.6%) | 5886 (36.8%) | 2483 (15.5%) | 1716 (10.7%) | 793 (5.0%) | 458 (2.9%) | 340 (2.1%) |
| Q5 | 8381 (52.4%) | 1683 (10.5%) | 6010 (37.6%) | 2616 (16.4%) | 1737 (10.9%) | 853 (5.3%) | 487 (3.0%) | 362 (2.3%) |
